# Cryo-EM Analyses Permit Visualization of Structural Polymorphism of Biological Macromolecules

**DOI:** 10.3389/fbinf.2021.788308

**Published:** 2021-12-08

**Authors:** Wei-Hau Chang, Shih-Hsin Huang, Hsin-Hung Lin, Szu-Chi Chung, I-Ping Tu

**Affiliations:** ^1^ Institute of Chemistry, Academia Sinica, Taipei, Taiwan; ^2^ Department of Applied Mathematics, National Sun Yat-sen University, Kaohsiung, Taiwan; ^3^ Institute of Statistical Science, Academia Sinica, Taipei, Taiwan

**Keywords:** strucural analysis, classification, crystal, solution, conformation alanalysis, heterogeneity, cryo-TEM, x-ray

## Abstract

The functions of biological macromolecules are often associated with conformational malleability of the structures. This phenomenon of chemically identical molecules with different structures is coined structural polymorphism. Conventionally, structural polymorphism is observed directly by structural determination at the density map level from X-ray crystal diffraction. Although crystallography approach can report the conformation of a macromolecule with the position of each atom accurately defined in it, the exploration of structural polymorphism and interpreting biological function in terms of crystal structures is largely constrained by the crystal packing. An alternative approach to studying the macromolecule of interest in solution is thus desirable. With the advancement of instrumentation and computational methods for image analysis and reconstruction, cryo-electron microscope (cryo-EM) has been transformed to be able to produce “in solution” structures of macromolecules routinely with resolutions comparable to crystallography but without the need of crystals. Since the sample preparation of single-particle cryo-EM allows for all forms co-existing in solution to be simultaneously frozen, the image data contain rich information as to structural polymorphism. The ensemble of structure information can be subsequently disentangled through three-dimensional (3D) classification analyses. In this review, we highlight important examples of protein structural polymorphism in relation to allostery, subunit cooperativity and function plasticity recently revealed by cryo-EM analyses, and review recent developments in 3D classification algorithms including neural network/deep learning approaches that would enable cryo-EM analyese in this regard. Finally, we brief the frontier of cryo-EM structure determination of RNA molecules where resolving the structural polymorphism is at dawn.

## Introduction

The concept of structural polymorphism has been associated with structure research in the beginning. In material science, structural polymorphism depicts the existence of a solid material in more than one form or crystal structure. Biological macromolecules are “soft material” that can readily change forms. We thus refer to “one biomolecule with multiple structures” as “structural polymorphism”. Proteins offer the best known example of biomolecule to show structural polymorphism. The tertiary structure of a protein can undergo minor or even global conformational changes depending on the intrinsic properties of the protein and external stimuli. Some proteins exhibit the capacity to rearrange the structures in response to an environmental trigger (e.g., pH, temperature/salt ions, small molecules, redox). Remarkably, the alteration of a local structure is capable of inducing the change of a remote site in the same molecule. Such “allosteric behavior” of a protein is not only germane to mechanistic understanding of many fundamental biological processes, but also to understanding of the function of agonists/antagonists in the field of pharmacology. Thus structural polymorphism in proteins is an area of great interests.

Proteins, arguably the most versatile macromolecules in living systems, serve crucial functions in virtually all biological processes. Made up of linear polymers with building units of amino acids, proteins can fold up into three-dimensional structures determined by the sequence of amino acids. Based on the structure-function paradigm ushered in by Anfinsen in the 60s, the function of a protein is directly dependent on its three-dimensional structure (Anfinsen and Haber, 1961). In late 50s Koshland hypothesized structural change of an enzyme at the active center (Levy et al., 1959), which was used to explain the temperature dependence of ATP hydrolysis rate by myosin (Koshland, 1958) and other enzymes in general. Eventually, this line of research had further expanded into a branch in enzymology on how changes in conformation would have function consequence for proteins to assume different functions or switch between inactive and active states with high specificity and affinity.

In the structural biology community, structural polymorphism of proteins can be loosely classified as either structural changes or dynamical changes. The former would involve different protein conformations (i.e. distinct ensemble of one protein subunit), protein configurations (i.e. distinct ensemble of a protein with multiple subunits), and protein states (i.e. equilibrium states such as open and close state of an ion channel) among others. The latter may include allostery (i.e. non-equilibrium based structural changes) and transition from one state to another. Recently it has been uncovered that intrinsically disordered proteins (IDPs) comprise a significant fraction of the proteome. These proteins exhibit polymorphic ensemble of conformations rather than a unique structure (Kulkarni et al., 2018). In any case, the above mentioned classification of structural polymorphism is perhaps phenomenological.

**FIGURE 1:**
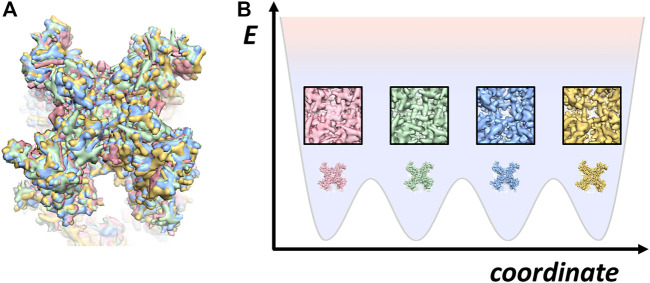
Snapshots of TRPV cryo-EM structures **(A)** four TRPV structures superimposed: apo followed by three different conformations bound with DkTx toxin (EMD-23136, PDB: 7L2P; EMD-23143; PDB: 7L2S; EMD-23141; PDB: 7L2T; EMD-23141; PDB: 7L2U) (Zhang et al., 2021). **(B)** a cartoon of energy landscape for illustrating the four different structures in **(A)**.

At fundamental level, structural polymorphism can perhaps be described using the theory of energy landscape (Frauenfelder et al., 1991). Proposed by Frauenfelder in the 70s to explain the complex kinetics observed from photolysis experiments on myoglobin (Austin et al., 1975), this notion of protein energy landscape connects structural polymorphism to thermodynamics where the manner of transitions among sub-states would be determined by the energy barriers (Figure 1).

However, visual evidence in support of multiple conformations from one protein had to come from X-ray crystal structure determination. The X-ray crystal diffraction approach with successful phasing provides observation of the electron density of an entire molecule to accurately define the positions of atoms in it. In X-ray crystallography, significantly different structures are largely derived from different crystal forms. Nonetheless, in a single crystal local motilities of a protein can be parameterized by temperature factors (B-factors) assigned to each atom when the resolution has reached 2.5 Å or beyond. These B-factors serve as indicators of the mobile chain segments, but cannot describe the exact motions during protein action (Vonrhein et al., 1995). Since most substantial chain displacements would affect packing contacts, the structural polymorphism could either destroy the crystals or change the crystal form─namely, a brand new structure often entails a new crystal form, but this new structure is again just another static structure (Schlichting et al., 1990). An example of structural polymorphism revealed by X-ray diffraction came from studies on the conformations of the enzyme adenylate kinase (AK). AK, ∼20 kDa in molecular weight, is a key signal transducing protein for controlling cellular energy homeostasis (Vonrhein et al., 1995) (see also reference therein). Heroic efforts in 1990’s on AK structure determination have produced a plethora of crystal structures (Vonrhein et al., 1995), demonstrating that protein conformational changes can be indeed drastic for this system (Schulz et al., 1990). Precisely, these conformational changes are characterized by the motions of internal chain segments relative to the main body constituted with the central parallel β-sheets (Vonrhein et al., 1995). These studies had set the quest for exploring the time trajectories of protein dynamics by single-molecule measurements (Roy et al., 2008; Tan et al., 2009) and molecular dynamics analysis (Formoso et al., 2015).

In this era of pandemic, it is important to recall early study on structural polymorphism of viral replication enzymes in relation to allostery by Peersen and his co-workers (Gong and Peersen, 2010). They assembled, purified, and crystallized poliovirus RNA-dependent RNA polymerase (RdRp) elongation complexes produced by multiple rounds of nucleotide incorporation. The crystal structures captured the active polymerase and its nucleotide triphosphate complexes in four distinct states, providing a working model describing the catalytic cycle of positive-strand RNA virus RdRps. Since RNA viruses encoding high- or low-fidelity RdRps are attenuated in the virulence, efforts were made to exploit the perturbed fidelity as the basis for rational drug design against RdRps. To do so, Hogle and Cameron (Moustafa et al., 2014) combined X-ray crystallography, spectroscopic and kinetic measurements to explain why some residues distant from the active site could affect the fidelity of nucleotide incorporation─they do so via altering the conformational dynamics of the active site.

Since its introduction in 1960s by Perutz (Perutz et al., 1968), X-ray protein crystallography had soon become the standard tool for determining the structure of proteins to atomic resolution. As the synchrotron light sources and computation software (Brünger et al., 1998) (see reference therein) were further advanced, the bottleneck of X-ray crystallography remains with crystallization. As mentioned earlier, a unique tertiary structure of a protein is likely to be output from a particular form of single crystal. In other words, a different structure would most likely come from a crystal of different symmetry or altered dimensions. This requirement of crystallization would thus impede easy access to structural polymorphism of biological macromolecules. More importantly, structural polymorphism obtained through X-ray crystallography is susceptible to criticism of not directly reflecting the functional states in solution. A notable case is yeast RNA polymerase II that manifests structural polymorphism (Fu et al., 1999; Cramer et al., 2000; Gnatt et al., 2001). As this polymorphism is associated with the swinging of a mobile domain involved in crystal packing, it was not clear at that time whether or not such polymorphism did bear direct physiological relevance. Therefore, there has been a cry for using an in solution approach to accessing the structure(s) of biological macromolecule.

**FIGURE 2:**
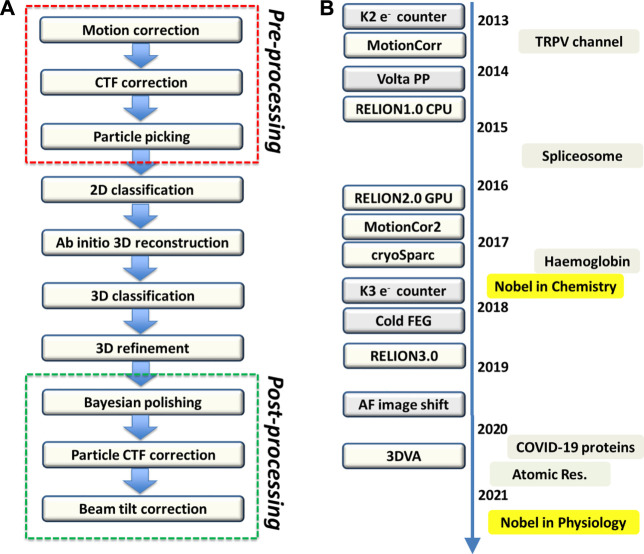
Single particle cryo-EM processing and milestones **(A)** Single particle processing. There are in general a total of 10 steps in the processing where the red box encloses the pre-processing steps, which can be executed in an on-the-fly manner. At the bottom, the post-processing steps encased by the green box are used to improve the overall resolution and local map quality as well. Bayesian polishing on RELION takes care of the dose weighting to compensate the frame-dependent radiation damage effect. Once a high-quality map is obtained, the variation of defocus and higher order aberration at per particle level can be further estimated and corrected. **(B)** Cryo-EM milestones. The boxes on the left of the timeline highlight key advancement of hardware and software where “AF image shift” stands for “aberration-free image shift.” The boxes on the right of the timeline indicate significant cryo-EM structures and events including recent rapid structure determination of COVID-19 proteins, achievement of atomic resolution with apo-ferritin, and the Nobel Physiology or Medicine in 2021 that recognizes the discoveries of TRPV and Piezo channels as heat and pressure sensors respectively where the structures were solely obtained by cryo-EM.

Two structure technologies designed for imaging the structures of radiation-sensitive materials in non-crystalline states would answer the call─ cryo-electron microscopy (cryo-EM) (Dubochet et al., 1988; Henderson, 1995; Frank, 2002) and free electron X-ray laser (XFEL) (Ekeberg et al., 2015). The usage of XFEL single particle diffraction imaging is mostly limited to giant particles whereas the high-resolution application still requires crystals of small size. Compared to XFEL, Cryo-EM directly obtained the projection density of the target macromolecule without the need of phase retrieval. Cryo-EM, in particular the single particle analysis (Figure 2A), has undergone resolution revolution to become a mainstream structural biology method (Kühlbrandt, 2014; Nogales and Scheres, 2015). Since biological macromolecule is radiation sensitive, cryo-EM is a dose-limiting imaging technique. In this regards, the cryo-EM resolution revolution has been critically dependent on the high-sensitivity of CMOS direct electron detector (Xuong et al., 2007; McMullan et al., 2009). Camera made of such sensors that can count electrons has greatly improved the quantum efficiency (see the milestones in Figure 2B). In addition, empowered by fast signal transfer rate, the direct electron CMOS cameras produce movie data of images with very large number of pixels. This capability allows for image motion correction to rescue the loss of image contrast due to charge-induced specimen movement (Brilot et al., 2012; Li et al., 2013). Furthermore, stable electron optics and high-quality vacuum has enabled continued data collection on microscopy without human intervention for days, which plays a key role in supplying high volume of data to suffice subsequent computation of structures to near atomic resolution. Due to the simplicity of sample preparation and high-efficiency in data collection using automated cryo-EM (Tan et al., 2016), single particle cryo-EM has rapidly gained popularity in structural biology and made impact on life sciences in general by initiating structure-guided function studies. In a short period of 3 years, the percentage of atomic models in the Protein Data Bank (PDB) derived from high-quality cryo-EM maps has surpassed 10% while this figure is rapid climbing.

As single particle cryo-EM relaxes the need on using crystalline materials, it immediately opens the door for structure determination of biological macromolecules that are refractory to crystallization (e.g. TRPV in the milestones in Figure 2B). Importantly, as the targeted macromolecules are now free from the constraint of crystal packing, connecting cryo-EM structures with functional states is straightforward and legitimate. A vivid example provided by cryo-EM is the structures of ATP synthases (Guo et al., 2017; Murphy et al., 2019) where multiple structural intermediates corresponding to the snapshots of the machine in action were captured.

Now, as all possible sampled states in solution or native conditions are all recorded by cryo-EM imaging, the image data are complex in nature. The data consists of an ensemble of different structures. Making a single 3D reconstruction of a macromolecule using all its cryo-EM images that represent the ensemble of co-existing structures would obviously yield a blurred structure of the macromolecule. To disentangle the co-existing structures, Scheres, Frank, and Carazo made a pioneering effort (Scheres et al., 2007) in modeling the data as a mixture of a number of discrete structures using a likelihood approach (Sigworth et al., 2010; Grant et al., 2018). This likelihood-based 3D classification approach later evolved to a Bayesian approach (Scheres, 2012). Those disentangling algorithms immediately contributed to the breakthrough in the structure determination of spliceosome particles (Yan et al., 2015) (see the milestones in Figure 2B), which have poised an impasse for crystallography as the dynamical remodeling has resulted in overwhelming heterogeneity.

Earlier applications of single cryo-EM structural determination of proteins tended to employ this tool as a “crystal-free” PDB structure generator. Researchers would check out a dominant structure from the image data with the resolution better than 3.5 Å to suit *de novo* model building while ignoring other structures co-existing in the data that were less prominent. Recently, there has been increased interest in extracting as many possible structures from one cryo-EM study, particularly for elucidating the action of COVID-19 spike protein (Yang et al., 2021). This trend has attested the demand on the potential of cryo-EM in uncovering structural polymorphism. In this review, we focus on representative cases on well-folded proteins with high significance to illustrate this development. We exclude the discussion on IDPs (Kulkarni et al., 2018) in this review since a disordered structure would not emerge through the image averaging process in cryo-EM analyses. In the end, we highlight the progress in 3D classification algorithms that resolve the structural polymorphism registered in the data, and discuss the possibility of visualizing RNA structural polymorphism by cryo-EM where the challenges are much greater than that of protein.

## Sub-100 kDa proteins

Since the high-resolution cryo-EM imaging is built upon phase contrast, visualizing small proteins is challenging. Considering proteins as small as adenylate kinase (AK), it could not generate sufficient signals under cryo-EM unless the targeted protein organizes into an oligomer. Currently, the smallest particles attainable by cryo-EM with near atomic resolution are represented by streptavidin (52 kDa) (Fan et al., 2019; Han et al., 2020) and haemoglobin (64 kDa) (Khoshouei et al., 2017; Herzik et al., 2019), both of which are in multimeric form.

**FIGURE 3:**
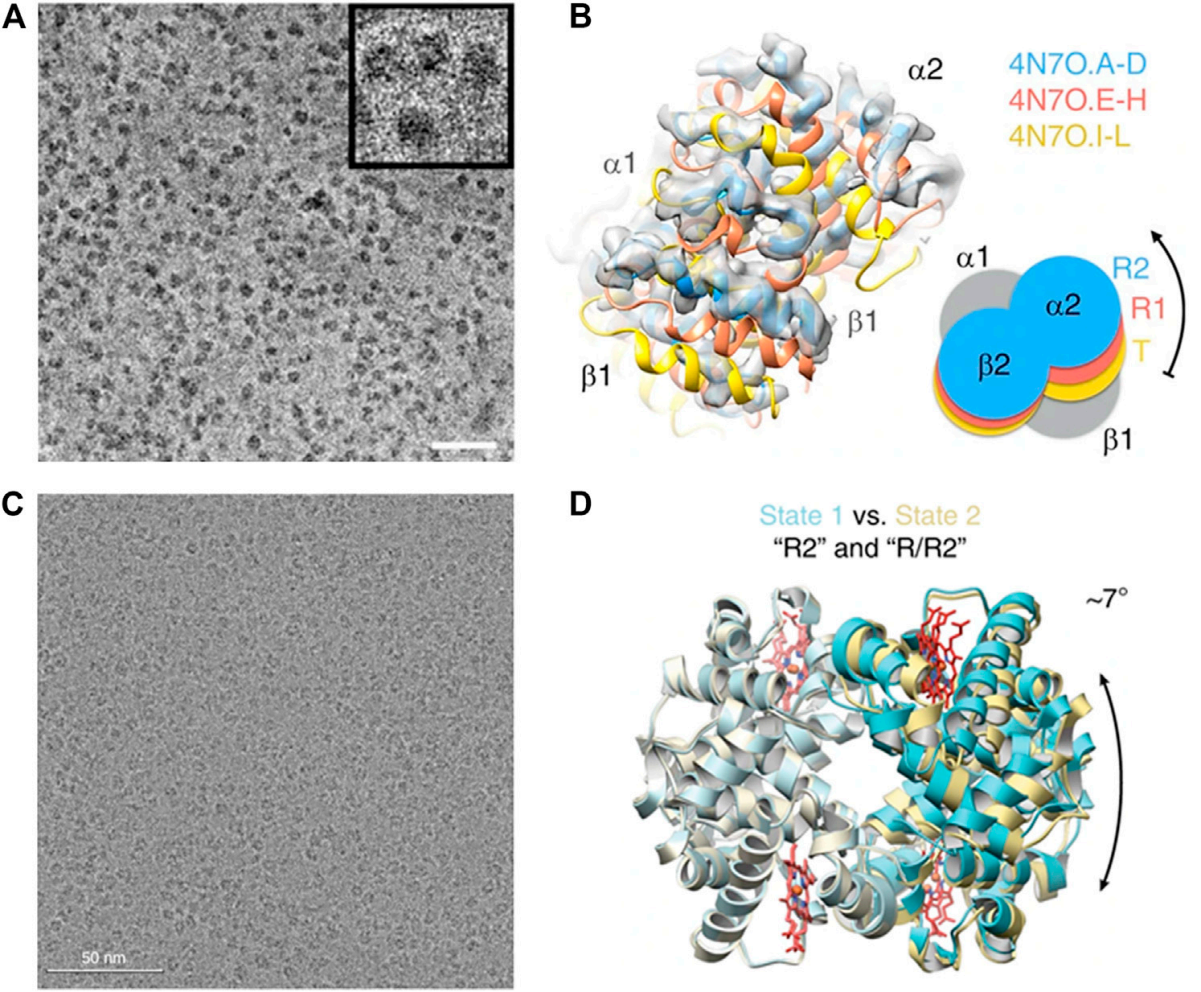
Single particle cryo-EM reveals co-exiting conformations of haemoglobin. **(A)** Visualization of haemoglobin with the aid of Volta phase plate (see the milestones in Figure 2B). (This figure is adopted from Figure 1A of Ref 41, an open access article distributed under the terms of the Creative Commons CC BY license. https://creativecommons.org/) **(B)** Modeling the cryo-EM map with known X-ray structures suggests the co-existing conformations. (This figure is adopted from Figure 1E of Ref 41, an open access article distributed under the terms of the Creative Commons CC BY license. https://creativecommons.org/). **(C)** Visualization of haemoglobin in thin ice without phase plate. This figure is adopted from Figure 1A of Ref 42. **(D)** Resolving the cryo-EM map with the usage of K-way 3D classification on RELION indicates the co-existing conformations. (This figure is adopted from Figure 1E of Ref 42, an open access article distributed under the terms of the Creative Commons CC BY license. https://creativecommons.org/).

Haemoglobin (Hgb) mediates oxygen transport in blood with four proteins organized as a dimer of αβ dimer in C2 symmetry; it is the first protein structure together with myoglobin solved using X-ray crystallography by the “isomorphous replacement” phasing technique,” invented by Max Perutz (Perutz et al., 1968). Almost 60 years later, the first cryo-EM image of haemoglobin (Hgb) was obtained by Danev and Baumeister (Khoshouei et al., 2017) (Figure 3A, adopted from Figure 1A in Khoshouei et al., 2017) with the usage of phase plate, which enables cryo-EM visualization of proteins smaller than 100 kDa (Chang et al., 2010; Wu et al., 2013). In the cryo-EM map of Hgb at 3.2 Å resolution, side-chain densities and prosthetic heme groups of Hgb are clearly resolved with C2 symmetry imposed (Khoshouei et al., 2017). The derived atomic model was compared with three conformers of ferrous (Fe^2+^) Hgb, tight (T), relaxed 1 (R1) and relaxed 2 (R2), obtained from the crystal structures by Shibayama et al. from Tame group in 2014 (Shibayama et al., 2014). Using rigid-body fitting to dock the α1 subunits, the authors found cross-correlation values of 43, 47 and 62% for T, R1 and R2 states, respectively (Khoshouei et al., 2017) (Figure 3B, adopted from Figure 1E in Khoshouei et al., 2017). This observation is consistent with that met-haemoglobin (metHgb), namely ferric (Fe^3+^) Hgb, can adopt an R-like state. It should be noted that this work did not invoke 3D classification analyses to disentangle the conformational sub-states that may co-exist.

A subsequent cryo-EM study on haemoglobin was performed by Lander and co-workers (Herzik et al., 2019) with the intention of demonstrating the imaging capability of conventional defocusing cryo-EM using optimized samples (Figure 3C, adopted from Figure 2A in Herzik et al., 2019). Remarkably, through extensive parallel 3D classification analyses on the conventional cryo-EM images of Hgb, two distinct conformational states of metHgb were revealed (Figure 3D, adopted from Figure 3C in Herzik et al., 2019). State 1 attaining 2.8 Å resolution closely resembles the R2 state described by the crystal structure of Shibayama et al. (PDB: 4N7P) (CαRMSD 0.4 Å) (Shibayama et al., 2014), and state 2 with 3.2 Å resolution matches well with the “between R1 and R2” state (PDB: 4N7N) (CαRMSD 0.5 Å) (Shibayama et al., 2014). Comparing the two states by superposition of a αβ dimer from each molecule indicated a less than 10° rigid-body rotation of one αβ dimer relative to the other with the rotation axis centered about the dimer–dimer interface. Those observations were consistent with those movements observed in the crystal structures (Shibayama et al., 2014). The latter cryo-EM study of haemoglobin with thorough classification analyses clearly demonstrates that high-resolution single-particle cryo-EM is applicable to resolve distinct, biologically relevant conformational states of a sub-100 kDa complex.

## Ion Channels

A variety of important cellular activities occur at the cell membranes including ion transport, signal transduction, and bioenergetics through the working of membrane proteins. Membrane proteins are notorious for crystallization. As a result, membrane protein crystal structures only comprise a small fraction in the PDB data bank. Ion channel, in particular, is a hard subject among membrane proteins as it cannot be easily prepared in large quantity because unwary over-expression would kill the host cells. Remarkably, as soon as the resolution of single particle cryo-EM was advanced with an electron counting camera (Li et al., 2013), Julius and Cheng immediately applied it to obtain a near atomic resolution structure of the ion channel of transient receptor potential (TRP) channel (Liao et al., 2013) with the protein stabilized by spider toxin and solubilized with detergent-like polymers.

**FIGURE 4:**
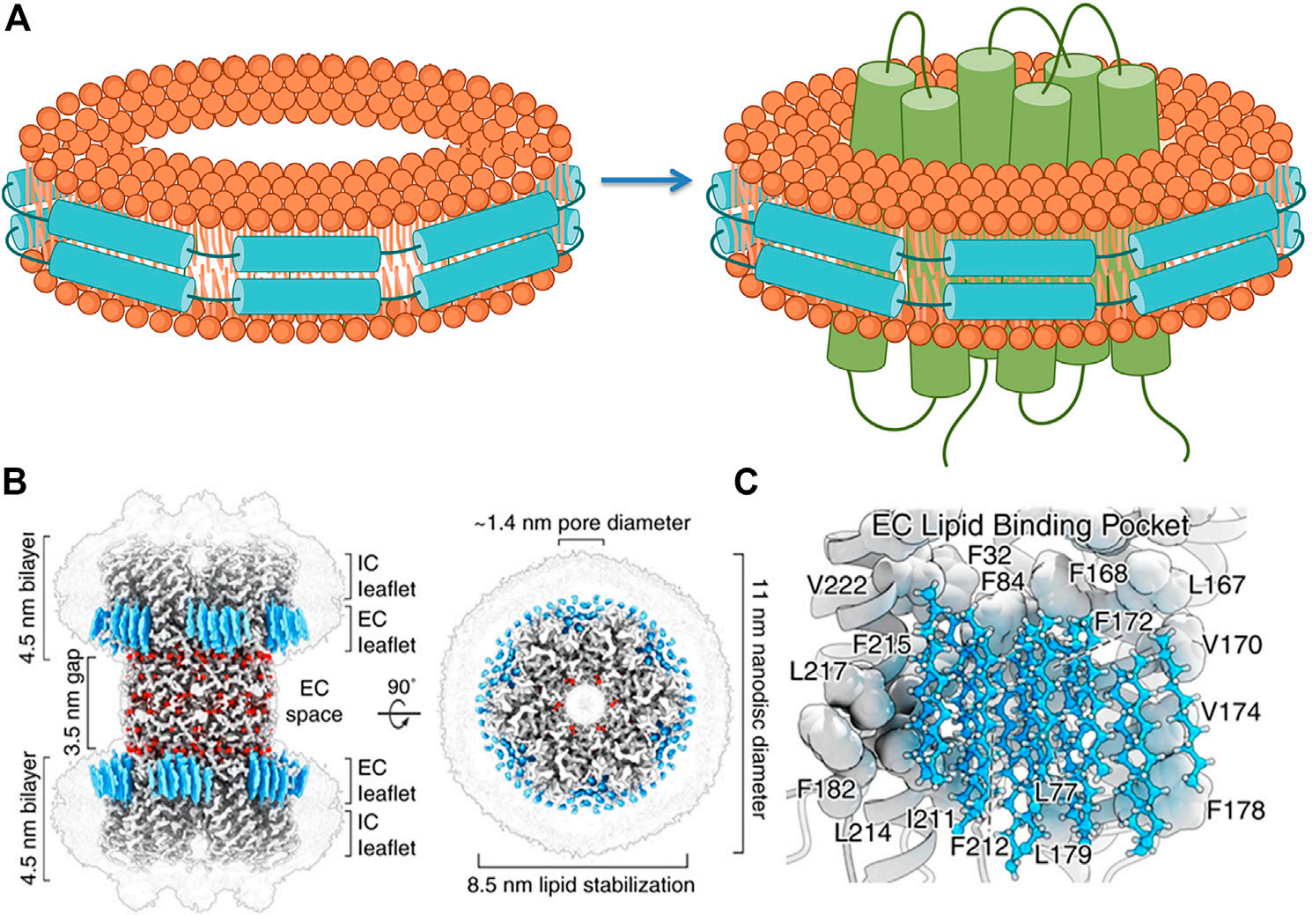
Nanodisc system facilitates cryo-EM analyses of membrane proteins in native environment. **(A)** Reconstitution of a nanodisc-membrane protein complex: lipids enclosed by membrane scaffold proteins (MSP) to form a nanodisc are assembled with a membrane protein to form a nanodisc-membrane protein complex. (This figure is made by modifying nanodisc cartoons available from BioRender with permission under a purchased license.) **(B)** Cryo-EM structure of a connexin protein in nanodisc. **(C) **Revelation of lipid-protein interactions **(B and C)** are adopted from Figures 1, 3 in Denisov and Sligar (2016) with permission from Creative Commons license).

Yet, membrane proteins are naturally embedded in lipid bilayers. To access the function and structure of membrane proteins in lipid bilayer, nanodisc was introduced by Sligar (Denisov and Sligar, 2016) (see Figure 4A). When a membrane protein is hosted in lipid bilayer encased by a scaffold protein that constitutes the nanodisc, it would allow the membrane protein in close-to-native environment to be characterized by NMR (Nasr et al., 2017) or visualized by cryo-EM (Flores et al., 2020) (Figure 4B, adopted from Figure 1 in Flores et al., 2020). Remarkably, as the resolution of cryo-EM imaging passes 2 Å, the protein-lipid interactions become visible (Flores et al., 2020) (Figure 4C, adopted from Figure 3 in Flores et al., 2020), demonstrating the possibility of seeing how lipids would participate in modulating the structure-function relationship of a membrane protein.

Among ion channels, TRP channels are peculiar as some families of these ion channels respond to diverse stimuli to allow passage of either small or large cations; this puzzle of functional plasticity has drawn great attention. Of particular interest is the presumable conformation dynamics associated with the ion selectivity filter regulated by physiological reagents. Over years, it has been speculated that there would be structural intermediates behind such dynamic channel regulation. But can they be captured? Considering TRPV1, time trajectories from channel recording suggested this channel would alter its ion selectivity in response to binding of a plethora of small molecules of algogenic agents. To investigate the corresponding structures, Cheng and Julius reconstituted TRPV1 into nanodisc to perform cryo-EM imaging on the channel protein triggered with various agents including protons, vanilloid agonists, and peptide toxins (Zhang et al., 2021). As opposed to the previous study of TRPV1 (Liao et al., 2013) that selectively stabilized a conformation using a spider double-knot toxin (DkTx) together with a vanilloid agonist resiniferatoxin (RTX), this study sought to assay the possible intermediate states of TRPV1 bound with DkTx alone, of which the mode of action is puzzling as it targets the outer pore region of TRPV1, but opens the lower gate to evoke membrane currents. Remarkably, the authors identified a collection of sub-states within the same cryo-EM dataset featured by distinct toxin interactions, pore diameters, coordination of ions by amino acids in the selectivity filter. A total of 26 high-resolution 3D maps of TRPV1 were presented in this milestone study (please see Supplementary Figure S1 in Zhang et al., 2021). Those maps represent the sub-states of apo-TRPV1 under standard conditions, TRPV1 bound with a spider toxin with varied stoichiometry, and apo-TRPV1 in various acidic conditions. The massive amount of structure information output by cryo-EM at once not only uncovers new findings to explain the function of TRP channels as polymodal signal integrators, but also demonstrates the efficacy of cryo-EM in rapidly revealing structural polymorphism of native membrane proteins. This capability of cryo-EM in visualizing key structural features of receptor/channel proteins including ligand-receptor interactions, binding site stoichiometry and cooperativity, and the mode of competition, would perhaps trigger a tsunami that impacts molecular physiology and pharmacology.

## Protein Machineries

Molecular machines are largely composed of large number of protein subunits, which may generate uncorrelated or correlated movements (Karplus and Kuriyan, 2005). GroEL, an Escherichia coli chaperonin (Hartl and Hayer-Hartl, 2002; Hayer-Hartl et al., 2016), is a molecular chaperone that assists the correct folding of proteins in the cell (Chaudhry et al., 2004); it is composed of 14 chemically identical protomers organized into two stacked seven-membered rings (see Hartl and Hayer-Hartl, 2002). Although the structure of GroEL has been previously characterized by both X-ray crystallography (Chaudhry et al., 2004; Bartolucci et al., 2005) and low-resolution cryo-EM in early days (Ludtke et al., 2004; Ludtke et al., 2008; Clare et al., 2012), those studies had applied D7 symmetry in the analysis of the structure by literally averaging over the protomers with assumption that they have identical conformations. In other words, those studies did not disclose any variations among the protomers in an oligomer.

**FIGURE 5:**
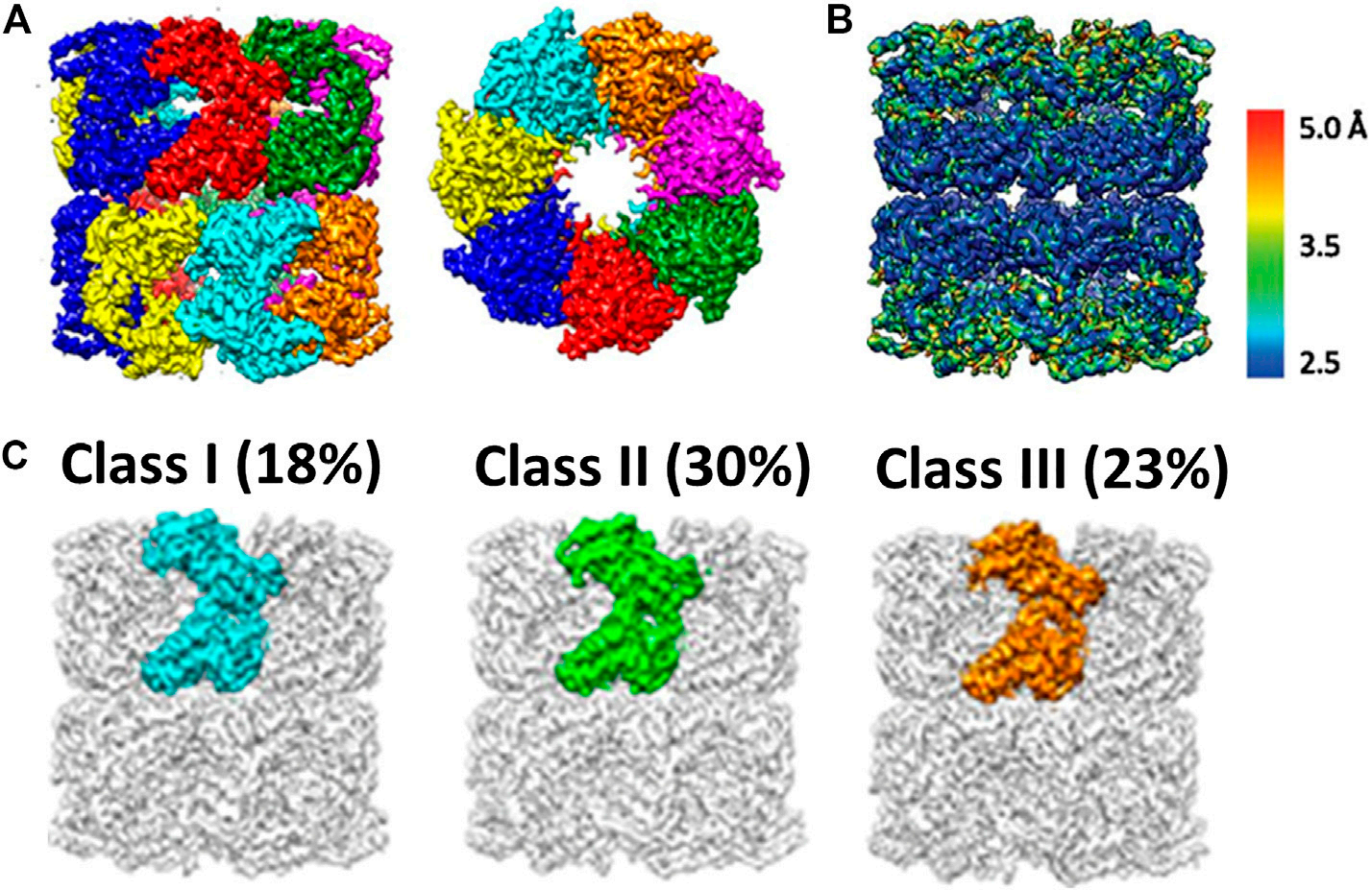
Cryo-EM analyses of GroEL, a multi-subunit protein machinery. **(A)** Segmentation of a high-resolution cryo-EM reconstruction. This allows extraction of individual subunit for further analysis. **(B)** Local Resolution map of GroEl. It shows the apical domain appears to be more dynamic as compared to equatorial domain. **(C)** Focused 3D classification on individual subunit. It reveals three major conformers (This set of figures is adopted from Roh et al (2017) with permission granted by NAS).

Even with the resolution breakthrough in single particle cryo-EM, it is not trivial to derive the atomic structures of individual subunits within a molecular machine of homo-oligomer such as GroEL because the task is extremely computationally expensive. The first report regarding the conformation heterogeneity of the promoters of GroEL was thereby not available until 2017. Chiu and coworkers (Roh et al., 2017) first reconstructed apo-GroEL to close to a resolution of about 3.5 Å from approximately 40,000 particle images. At this resolution, 14 individual protein subunits were unambiguously isolated and segmented (Figure 5A, adopted from Figure 1C in Roh et al., 2017). In contrast to X-ray crystallography, cryo-EM structures provide information of local resolutions (Kucukelbir et al., 2014) in the maps in addition to a mean resolution. Local resolution analyses of the GroEL cryo-EM structure revealed that the apical domain was resolved to ∼4 Å and the equatorial domain to 3 Å (Figure 5B, adopted from Supplementary Figure S3 in Roh et al., 2017). This observation implied greater flexibility of the apical domain and suggested that structural variations may well exhibit among the 14 compositionally identical subunits of a GroEL oligomer. Since a naïve 3D reconstruction of GroEL without imposing symmetry would still treat the particle as a whole object, it may not be sensitive to subtle differences within a particle that vary from particle to particle, in particular in the “sea” of signals from an entire GroEL oligomer. Therefore, Chiu and coworkers forged a computationally expensive task of invoking focused 3D classification introduced by Scheres (Bai et al., 2015), and also independently by Huiskonen (Ilca et al., 2015)─in this approach, individual subunit in each particle image was computationally extracted, by which distinct conformations, if any, could be detected and resolved. This analysis allowed for sorting the subunits into a large number of classes based on the conformational similarity─approximately 70% of the subunits were categorized into three major conformation classes (Figure 5C, adopted from Figure 3 in Roh et al., 2017). The primary difference among the three conformers is localized to the apical domain, involved in substrate binding, where its orientation relative to the equatorial domain varies due to the movement of the intermediate domain that connects them. Interestingly, the spatial distributions of each conformation class differed among GroEL oligomers, with most oligomers containing 10–12 subunits in one of the three major conformations. In addition, correlation among subunits seems to exist in a GroEL oligomer as adjacent subunits were found to assume the same conformation. It is noted those three conformations match well with those found in GroEL X-ray structures, leading to the conclusion that the structural polymorphism previously observed in the crystal structures were not a crystallization artifact.

When chaperons are in action, they capture non-native polypeptides in the central cavity and rectify their folding with the fuel of ATP (Zhao et al., 2021). Kinetic measurements indicated ATP hydrolysis by such oligomeric complex exhibited both positive and negative cooperativity (Yifrach and Horovitz, 2000; Kafri et al., 2001; Kusmierczyk and Martin, 2003). The question is raised as to what would be the structure basis underlying the cooperation of subunits in ligand binding and/or catalysis. In the case of group I chaperons to which GroEL belongs, intra-ring conformational changes are thought to follow a concerted, Monod–Wyman–Changeux (MWC) model (Yifrach and Horovitz, 1995; Dyachenko et al., 2013; Horovitz, 2013). In this model, each ring is in equilibrium between two states that can interchange in an orchestrated manner. This is because there are steric repulsions between subunits in GroEL that conformation changes in individual subunits are dis-allowed except when the concerted intra-ring transition occurs (Ma and Karplus, 1998; Ma et al., 2000). In contrast, in group II chaperonins, as the open conformation exhibits little contacts between the intermediate or apical domains of neighboring subunits, there is no hindrance to prohibit independent conformation changes in individual subunits (Huo et al., 2010; Zhang et al., 2010; Yébenes et al., 2011; Zhang, 2011). This means that the cooperativity in MmCpn, an archaeal chaperonin complex that belongs to group II may follow a different intra-ring allosteric mechanism.

To test whether nucleotide binding to one subunit increases the probability of binding to an adjacent subunit more than remote subunits in the same particle of MmCpn, Zhao et al. (Zhao et al., 2021) used single particle cryo-EM with the focused 3D classification analysis to assess the number and the distribution of nucleotides bound to each subunit in this homo-oligomeric complex. As shown in Figure 3 in Zhao et al., 2021, eight major classes representing distinct sub-conformations are obtained with the distal tip of the apical domain moving inwards ∼35 Å between the most open conformation (class 1) and the most closed conformation (class 8). To assess the nucleotide occupancy in the 8 sub-conformations, difference mapping was performed. Interestingly, nucleotide density is present in the four more-closed conformations (class 5–8), but is absent in the four more-open conformations (class 1–4), consistent with the notion that the nucleotide binding and/or hydrolysis can cause the conformation of subunit to switch from the open to the closed form (Sekiguchi et al., 2013). Next, Zhao et al. further extended the analysis on individual ring of each particle so as to label each of the 8 subunits as apo- or ATP-bound according to the 3D class they belonged to. This way the experimental observations of the relative frequency distribution of nucleotide occupancy at a subunit level within a ring were obtained, and then compared to those based on that predicted from random binding (please see Figure 4B in Zhao et al., 2021). The remarkable quantitative agreement concluded that ATP binding to subunits in the case of MmCpn is stochastic, which is expected as the conformational change induced by ATP binding of a subunit is unfettered by inter-subunit interactions within a ring.

## Temperature-Gated Cryo-Electron Microscope Studies

Those highlighted cases of structural polymorphism either stem from conformational sub-states associated with the protein dynamics or represent an outcome induced by ligand binding. The aforementioned samples used for cryo-EM studies, like nearly all other cryo-EM studies, were equilibrated at 4°C prior to plunger freezing, which is a common practice in cryo-EM community. As raising temperature can increase protein dynamics and 4°C is much far away from the point where most of enzymes are catalytically active, it is likely that the structures obtained from those cryo-EM studies may not closely reflect those of the macromolecules in action. To address this issue Chang et al. (Chang et al., 2021) modified the Vitrobot freezing device to enable the investigation of the structure of ketol-acid reductoisomerase (KARI) found in a hot spring bacteria (Sulfolobus acidocaldarius) that exhibits optimal enzyme activity in the range of 60–70°C (Chen et al., 2018). By clamping the temperature of vitrification at various temperatures ranging from 4 to 70°C, high-resolution structures of the enzyme at different temperatures were obtained (Chen et al., 2019). As shown in Figure 4 in Chen et al., 2019, the enzyme conformations remain largely unchanged in the range of 4–55°C. Strikingly, a major change was observed as the temperature was shifted to 70°C. The temperature-resolved structures seem to correlate well with the temperature-dependent activities. This study offers opportunities for dissecting the induced-fit mechanism by separating the contribution of ligand-induced from that of temperature-induced. Importantly, it demonstrates that cryo-EM can be used to obtain protein structures at functional temperatures. Along this line, a recent study of TRPV1 at elevated temperature has revealed the snapshots of heat-dependent opening of TRPV1 in the presence of capsaicin that underlies the mechanism of nociception (Kwon et al., 2021).

## Discussion

In this focus review, we choose a few milestone cases of cryo-EM structures including sub-100 kDa proteins, membrane proteins, and protein machineries to illustrate the exciting frontier of using cryo-EM to visualize structural polymorphism of protein complexes. In the past, structural polymorphism was best approached by crystal studies, but the physiological relevance of those structures were often in question due to the potential artifact induced by crystal packing. Beyond removing crystal-associated artifact, cryo-EM opens a chapter for structural studies of biological macromolecules that are refractory to crystallization. In the case of TRPV channels, those ion or ligand induced polymorphism is largely inaccessible by X-ray crystallography as membrane proteins often resist crystallization. As the complexity increases, application of cryo-EM to protein machines of oligomeric subunits, as exemplified by GroEL, other chaperons, and proteasomes, has uncovered the spatial distribution of ligand occupancy to portray individual machine as “single molecule” and provide structure basis for the cooperativity models inferred from bulk biochemistry measurements. This new opportunity is perhaps completely outside the reach of crystallography, because crystallization of protein machine like chaperon would perhaps trap it in a unique configuration permitted by symmetry.

Though the opportunity of structural polymorphism has been permitted by cryo-EM in the beginning, conventional usage of cryo-EM since the resolution revolution mostly employs it as an alternative approach to X-ray crystallography in deriving a PDB model for a protein. In this classical approach, the role of 3D classification analyses is largely aimed to “purify” a subset of high structural homogeneity from the cryo-EM data to produce a density map of best quality or highest resolution. As such, researchers may discard other 3D reconstructions also uncovered from the data that were less prominent. However, there is a shift of focus that is taking place. Considering TRPV channel that has been crowned with Nobel Prize of Physiology or Medicine 2021, since its first cryo-EM structure was determined in 2013 (Shibayama et al., 2014), significant improvements in sample preparation, cryo-EM data collection and analyses have been made. Those advancements have transformed our capability of exploring structural sub-classes of nanodisc-embedded TRPV to shed lights into the mechanism.

So far, virtually all the cited examples were analyzed with the standard K-way 3D classification method using Relion (Scheres, 2012). Although the K-way 3D classification is effective for checking out a consensus image subset that is structurally homogeneous, it is limited to resolving discrete structural heterogeneity. Therefore, there has been continued interest in developing 3D classification methods for analyzing continuous conformations pertinent to the dynamics of the target systems since early days of single particle cryo-EM. When an approach can accommodate continuous motions, it has the potential to capture transient dynamics of the system of interest. This effort is perhaps crucial in establishing the cause and effect relationship between the SARS-Cov2 spike mutants (Yang et al., 2021) and gain- or loss-of-function in its infectious activities.

**FIGURE 6:**
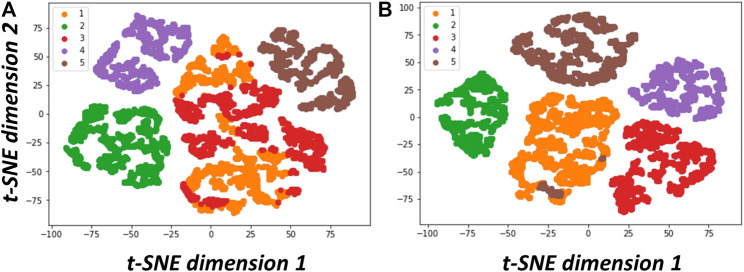
Data visualization of structural variability resolved via PCA analyses. **(A)** t-SNE plot of the results from classical PCA (Penczek et al., 2011). **(B)** t-SNE plot of the results from two-stage PCA (2SDR) (Chung et al., 2020). As stated in Chung et al., 2020, We followed the procedure in Penczek et al., 2011 to generate a dataset containing 9,453 simulated cryo-EM particle images projected from five 70S ribosome conformations with minor differences resulting from combinations of the absence or presence of tRNA (transfer RNA) and EF-G (elongation factor G). We then resampled these particle images to generate 11,000 3D volumes (density maps) on 75 × 75 × 75 voxels. Next, we solved the eigenvolumes using PCA (Penczek et al., 2011). or 2SDR (Chung et al., 2020). and compared the performance of these two methods using the factorial coordinates defined in Penczek et al., 2011. The t-SNE plots and k-means with 5 classes on their factorial coordinates indicate that the eigenvolumes solved by 2SDR have clearly resolved the structural variability (This set of figures is adopted from Chung et al., 2020 with permission given by International Press.).

We list in Table 1 concurrent 3D classification approaches developed for resolving structural heterogeneity and make comments based on our hands-on test experience. They can be roughly categorized into 1) classical dimension reduction or linear sub-space modeling approach (Penczek et al., 2011; Tagare et al., 2015; Andén and Singer, 2018; Chung et al., 2020; Chung et al., 2021; Punjani and Fleet, 2021a), 2) novel manifold embedding technique with non-linear sub-space modeling (Dashti et al., 2020), and 3) artificial intelligence-based approach (Zhong et al., 2021; Chung et al., 2021; Punjani and Fleet, 2021b). The dimension reduction approach is best featured by principal component analysis (PCA eigen-analysis) that was developed earlier on (Penczek et al., 2011). The PCA-based methods also have the capacity of modeling both discrete conformations in addition to continuous conformations. In the case of discrete states, the number of states can be visualized in a plot of the space of eigenvectors (Figure 6 adopted from Figure 2 in Chung et al., 2020), where the eigenvectors often correspond to features providing biological insights. However, earlier algorithms developed for this approach were all heavy in computation as they invoked bootstrap large number of 3D reconstructions from random subsets of images or entailed solving covariance matrix of high dimension where the dimension grows with targeted resolution. Currently, there is active development to reduce the complexity from high dimension (Andén and Singer, 2018) whereas a stage-wise dimension reduction approach (Chung et al., 2020) has been successfully applied to experimental cryo-EM images. There is also novel approach within this framework that circumvents the need of solving covariant matrix (Chung et al., 2021). Due to the limitation by the issue of dimension, those PCA methods would be limited to solving heterogeneity at low resolution when computation resources are modest. It is noted that, 3DVA (Punjani and Fleet, 2021a), a recently released approach, enables fitting high-resolution linear subspace models to single particle cryo-EM data and has been demonstrated effective for resolving continuous flexibility. As 3DVA is implemented on cryoSparc (Punjani et al., 2017), another package as popular as Relion, it has received wide attention in the cryo-EM community to make immediate impact on COVID-19 research (Yang et al., 2021). It is also noted that a PCA approach developed by Stark group with implementation on COW has been available (Haselbach et al., 2017) for facilitating investigation on conformation dynamics (Haselbach et al., 2017) or energy landscape (Haselbach et al., 2018). For the latter case, allosteric regulation of human holo-proteosome was revealed (Haselbach et al., 2018). To reconstruct energy landscape, ManifoldEM (Dashti et al., 2020), developed by Ourmazd for analyzing cryo-EM images and XFEL diffraction images as well, employed manifold embedding and non-linear subspace mapping to position each cryo-EM image of a macromolecule on a complex energy surface. This tool allows the entire work-cycle of a molecular machine to be visualized as it passes through a continuum of states. However, it is challenging to endow the manifolds with biological meaning, while interpreting the structures on the manifold is time-consuming. In addition, to perform ManifoldEM analysis involves many sub-steps where the parameters at each step have to be fine-tuned.

**TABLE 1:** Features of various 3D classification methods.

**Method**	**Strengths**	**Shortcomings**
RELION (Grant et al., 2018) ^(Feature:^ ^*C*)^	1. Most popular	1. Discrete structures only
	2. Enabled by a pipeline	2. The number of discrete states is to be determined manually
Eigen-analysis (PCA) (Penczek et al., 2011; Tagare et al., 2015; Andén and Singer, 2018; Chung et al., 2020; Punjani and Fleet, 2021a; Chung et al., 2021) ^(Feature:^ ^*V*)^	1. Continuous structures with interpretable eigen bases	1. Heavy computation complexity since it needs to compute the 3D covariance matrix explicitly, and limited in resolution
	2. Can relate to energy landscape. (Haselbach et al., 2017)	2. Restrict the conformation change to a linear combination of eigen bases
3DVA (Punjani and Fleet, 2021a) ^(Feature:^ ^*V*)^	1. Continuous structures with interpretable eigen bases	1. Heavy computation complexity in solving eigen bases
	2. Through an expectation-maximization approach, there is no need to solve the 3D covariance matrix	2. Restrict the conformation change to a linear combination of eigen bases
3DFlex (Punjani and Fleet, 2021b) ^(Feature:^ ^*V*,^ ^*N*)^	1. Continuous structures available	1. Use an auto-decoder model, which is computationally expensive
	2. Directly model the motion rather than model the 3D density	2. The relative distances in the conformational space are arbitrary that it is hard to relate to the energy landscape
CryoDRGN (Zhong et al., 2021) ^(Feature:^ ^*V*,^ ^*N*,^ ^*C*)^	1. Continuous structures available	1. Long training time required
	2. It does not need a 3D initial model and particle orientation parameters	2. Limitation in interpreting the reconstructed dynamic structure since no eigen base is available
e2gmm (Chen and Ludtke, 2021) ^(Feature:^ ^*V*,^ ^*N*,^ ^*C*)^	• Ease in the interpretation of the motions by comparing the Gaussian model generated at different positions of conformation space	• Resolution and size of the structure are limited due to the large memory usage for representing the 3D Gaussian mixture model^a^.
ManifoldEM (Dashti et al., 2020) ^(Feature:^ ^*V*,^ ^*C*)^	1. Continuous structures available	1. Long computational time with many tuning parameters in each stage
	2. Energy landscape directly available with a rigorous theoretical foundation	2. The underlying mathematics tools are drawn from many sub-disciplines and not easy to understand

V: data visualization; **N**: Neural network-based; C: code available.

a Directly quoted from Chen and Ludtke, 2021: “GPU memory currently limits the size and resolution of the model. For example, a GPU with 11 Gb of memory supports up to 3,200 Gaussians with particles sampled at 128 × 128 pixels, and a batch size of eight during training. This would be sufficient to represent the 50S ribosome at a roughly 8Å resolution, or smaller proteins at proportionally higher resolution. So, for many proteins, the method is currently limited to variations at the level of secondary structural features. This limitation is due to the Gaussian representation currently required by the underlying TensorFlow system.”

Recently, there is a surge of neural network or deep learning based approaches to 3D classification, to name a few, CryoDrgn (Zhong et al., 2021), e2gmm (Chen and Ludtke, 2021), and 3DFlex (Punjani and Fleet, 2021b), where the codes have been released for usage except 3DFlex. CryoDrgn uses deep neural network to directly reconstruct continuous distributions of 3D density maps and map the variability at per-particle level of single-particle cryo-EM datasets. Also using deep leaning architect, e2gmm determines a conformational landscape for proteins using a 3D Gaussian mixture model mapped onto 2D particle images in known orientations; it can automatically resolve the structural heterogeneity within the protein complex and map particles onto a small latent space describing conformational and compositional changes. Compared to CryoDrgn, e2gmm is relatively faster, more intuitive, and easier to use as it is implemented on EMAN2. However, the attainable resolution with e2gmm strongly depends on the available computation resources (see the comment made by the authors in Table 1). The above-mentioned neural network approaches identify structural heterogeneity including conformation dynamics at the level of 3D reconstruction/3D classification. By contrast, DeepMap (Matsumoto et al., 2021), is a newly developed neural method that can work on already obtained 3D cryo-EM map of a static structure to uncover the hidden dynamics. Conventionally, such hidden dynamics has to be acquired experimentally using NMR, hydrogen-deuterium exchange mass-spectroscopy (HDX-MS), or computationally with molecular dynamics simulation on the structure. Remarkably, DeepMap can access to the dynamics of extremely large systems including virus particles and large protein complexes that cannot be solved using those conventional techniques. Of note, the neural network approach is now being actively developed for nearly all steps in cryo-EM image analysis workflow including micrograph de-noising (Tegunov and Cramer, 2019; Bepler et al., 2020; Palovcak et al., 2020) for particle picking, selecting good 2D classes (Li et al., 2020), 3D alignment (Jiménez-Moreno et al., 2021), and even for 3D map masking and sharpening (Sanchez-Garcia et al., 2021). Yet a user must bear in mind that any neural network approach would be time-consuming.

As there is increased interest in resolving structural heterogeneity, the demand on collecting more image data is likely to increase. To reconstruct a high-resolution single cryo-EM structure of low or no symmetry to near atomic resolution usually requires 10^5^ particles of nearly homogeneous structure. This structure usually represents the dominant state that comprises a significant fraction of the image data whereas the remaining other structures corresponding to the less populated states would contain rich information as to structural polymorphism. In order to increase the map quality of those remaining sub-states, the size of dataset has to be enlarged, usually by many folds to an order of magnitude. With automated cryo-EM models such as Titan Krios and cryo-ARM, the current benchmark of throughput of data collection enabled by an imaging scheme of aberration-free image shift (Wu et al., 2019; Efremov and Stroobants, 2021), has reached a figure greater than 5,000 micrographs per day (Wu et al., 2019; Zhang et al., 2020). As a result, it is now possible to obtain sub-million to a million of particles on daily basis to afford the data volume to support fruitful mining on structural polymorphism. The drastic increase of the data volume would further burden the image analysis. Therefore, there will be expectation on developing efficient 3D classification algorithms so as to accelerate the rate of structure discoveries.

In this review, we mainly focus on structural polymorphism on well-behaved proteins and exclude those disease-associated amyloid proteins that are highly polymorphic in structures (Kollmer et al., 2019). Apart from proteins, RNAs are functional biological macromolecules─they fold into tertiary structures to exercise specific biological functions in addition to carrying genetic information coded in the sequence. Structural studies of RNA-only molecules have been extremely challenging with conventional structural methods such as X-ray crystallography and NMR. In the PDB, there are only ∼1,000 RNA-only structures compared to more than 10,000 protein structures since RNAs are refractory to crystallization. This issue perhaps traces its root to the extreme conformational heterogeneity inherent with RNA. A single strand RNA is prone to produce different sets of secondary structures that have comparable free-energy (Low and Weeks, 2010; Wang et al., 2021). Once the secondary structures fold into a tertiary structure, it would readily change conformations.

Despite that cryo-EM has made breakthroughs in high-resolution structural determination of large RNA-protein complexes previously unattainable by X-ray crystallography or NMR, a general view in the community of structural biology is that cryo-EM is ill-suited for most RNA molecules in the absence of protein partners as RNAs were too small or too flexible (Kappel et al., 2020). The developments of cryo-EM structure determination on RNA-only has thus been halted. Now, considering proteins again, in particular those without no or low symmetry, high-resolution cryo-EM determination is limited to 60 kDa, This would set the size of RNA-only molecules feasible with cryo-EM to be roughly 200 nucleotides (Kappel et al., 2020). Unfortunately, many functional noncoding RNAs are of similar size or smaller and thereby excluded from the reach of cryo-EM. Fortunately, as cryo-EM map describes the electrical potential of atoms, the small potential from RNA phosphate backbone allows nucleotide bases to be readily distinguished at moderate resolution (4 Å) (Wilkinson et al., 2018). Despite that at this resolution, purines (A, G) can be discerned from pyrimidines (U, C); only when the resolution is better than 3 Å, can adenosine be further distinguished from guanosine bases (Wilkinson et al., 2018). In other word, the resolution criteria required for modeling RNA from *De Novo* is more stringent than that for protein (Cheng, 2015).

To explore the potential of cryo-EM on RNA, Chiu and his colleagues initiated cryo-EM studies on RNA-only molecules since 2018. They tested a total of 18 functionally diverse RNAs with the size in the range 65–388 nucleotides (21–126 kDa) including Tetrahymena ribozyme, of which the complete structures were then unknown (Kappel et al., 2020). In order to enhance the visibility of those small RNAs, phase plate imaging technique was employed. The testing results show cryo-EM could resolve global folds of RNA molecules. Since the resolutions of the attained cryo-EM map (>4.7 Å) were not sufficient for *De Novo* model building, a pipeline (Ribosolve) that performs computer modeling by combining cryo-EM map and sequence data was developed for the structure determination of RNA molecules. Subsequently, Tetrahymena ribozyme in full-length was selected for the pursuit of high-resolution structure determination to reach 3.1 Å (Su et al., 2021). This result perhaps represents the RNA of highest resolution solved by cryo-EM. Concerning the potential of resolving structural polymorphism, there were a number of ribozymes and riboswitches together with human U1 snRNA were not pursued for 3D reconstruction as they could not generate meaningful 2D class averages (see Figure 2 in Kappel et al., 2020). Of particular interest is V. cholera glycine riboswitch. Using the K-way 3D classification, the authors have resolved at least four structurally distinct classes where the dominant group was further pursued to generate a cryo-EM map of 4.8 Å (see Extended Data Figure 3 in Kappel et al., 2020). These observations imply the existence of trackable structural polymorphism that merits further investigation. In conclusion, those recent exciting developments on RNA structures by cryo-EM have heralded new era of RNA structural biology where solving the issue of polymorphism related to its folding or dynamics is at dawn.
